# Supplementary material for: Sex Disparities in the Direct Cost and Management of Stroke: A Population-Based Retrospective Study
Source: Healthcare (Basel). 2024 Jul 9;12(14):1369. doi: 10.3390/healthcare12141369 (PMC11275613; doi:10.3390/healthcare12141369)
Supplement: Supplementary file 1 [file healthcare-12-01369-s001.zip › healthcare-3020509-supplementary.pdf]

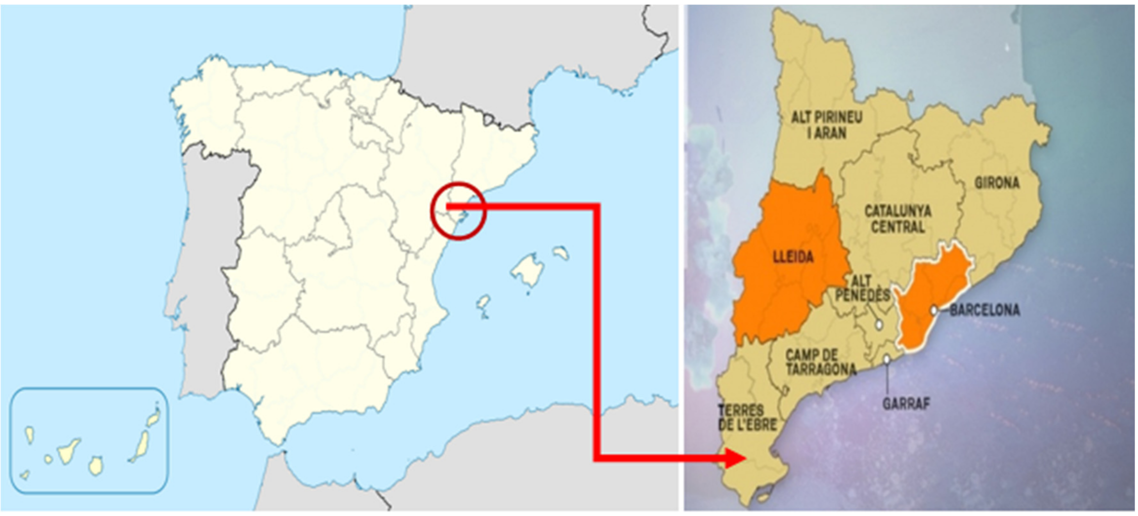

Figure S1. Terres de l'Ebre (RSTE), south Catalonia, Spain (2023).

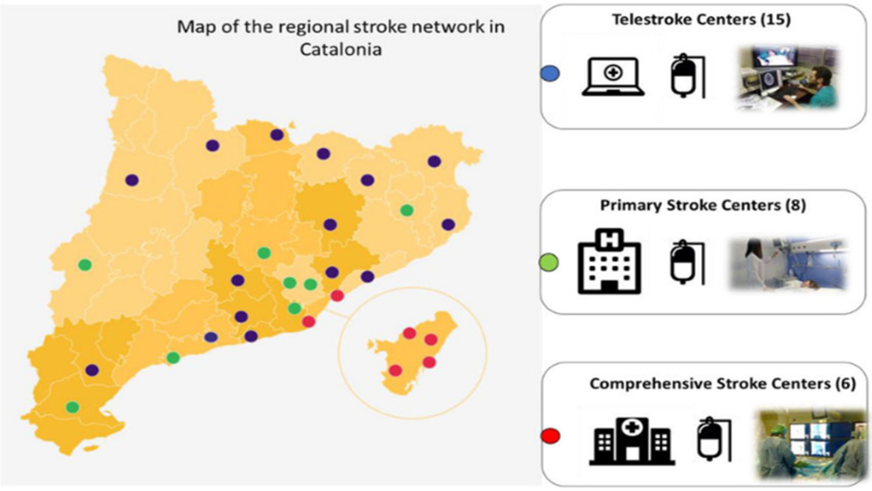

Figure S2. Stroke Code network Catalonia, in which TEV is available [21].

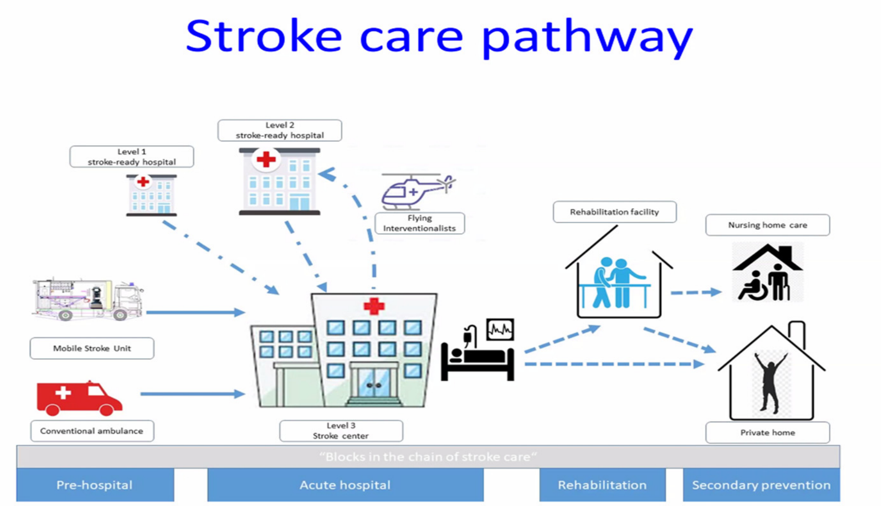

Figure S3. Stroke care pathway.

Table S1. STROBE Checklist.

STROBE Statement—Checklist of items that should be included in reports of ***cohort studies***

	Item No	Recommendation
**Title and abstract**	1	(*a*) Indicate the study's design with a commonly used term in the title or the abstract<br>Observational study as stated in the abstract (Page 1).
		(*b*) Provide in the abstract an informative and balanced summary of what was done and what was found<br>Provided in Abstract (Page 1)
**Introduction**		
Background/rationale	2	Explain the scientific background and rationale for the investigation being reported<br>Included in the introduction on pages 2 and 3.
Objectives	3	State specific objectives, including any prespecified hypotheses<br>Included in the introduction on page 3.
**Methods**		
Study design	4	Present key elements of study design early in the paper<br>Included in the Materials and Methods on page 3.
Setting	5	Describe the setting, locations, and relevant dates, including periods of recruitment, exposure, follow-up, and data collection<br>Included in the Materials and Methods on page 3.
Participants	6	(*a*) Give the eligibility criteria, and the sources and methods of selection of participants. Describe methods of follow-up<br>Included in the Materials and Methods on pages 3 and 4.
		(*b*) For matched studies, give matching criteria and number of exposed and unexposed<br>Not applicable.
Variables	7	Clearly define all outcomes, exposures, predictors, potential confounders, and effect modifiers. Give diagnostic criteria, if applicable<br>Included in the Materials and Methods on pages 5 and 6.
Data sources/ measurement	8*	For each variable of interest, give sources of data and details of methods of assessment (measurement). Describe comparability of assessment methods if there is more than one group<br>Included in the Materials and Methods on pages 4, 5 and 6.
Bias	9	Describe any efforts to address potential sources of bias<br>Described in the Strengths and limitations on pages 19 and 20.
Study size	10	Explain how the study size was arrived at<br>Included in the Materials and Methods on page 3 and 4. (The sample was derived from patients diagnosed with stroke in the period and territory described).

Quantitative variables	11	Explain how quantitative variables were handled in the analyses. If applicable, describe which groupings were chosen and why<br>Described in the Materials and Methods on page 6.
Statistical methods	12	(*a*) Describe all statistical methods, including those used to control for confounding<br>Described in the Materials and Methods on page 6.
		(*b*) Describe any methods used to examine subgroups and interactions<br>Described in the Materials and Methods on page 6.
		(*c*) Explain how missing data were addressed<br>Described in the Materials and Methods on page 6.
		(*d*) If applicable, explain how loss to follow-up was addressed<br>Not applicable
		(*e*) Describe any sensitivity analyses<br>Not applicable
**Results**		
Participants	13*	(a) Report numbers of individuals at each stage of study—eg numbers potentially eligible, examined for eligibility, confirmed eligible, included in the study, completing follow-up, and analysed<br>Described in the Results on page 7.
		(b) Give reasons for non-participation at each stage<br>Not applicable.
		(c) Consider use of a flow diagram<br>Not required.
Descriptive data	14*	(a) Give characteristics of study participants (eg demographic, clinical, social) and information on exposures and potential confounders<br>Described in the Results on page 7.
		(b) Indicate number of participants with missing data for each variable of interest<br>Not applicable.
		(c) Summarise follow-up time (eg, average and total amount)<br>Not applicable.
Outcome data	15*	Report numbers of outcome events or summary measures over time<br>Not applicable.
Main results	16	(*a*) Give unadjusted estimates and, if applicable, confounder-adjusted estimates and their precision (eg, 95% confidence interval). Make clear which confounders were adjusted for and why they were included<br>Included in the Results on pages 7,10,11,12 and 16. Summarized in tables 1 and 2.
		(*b*) Report category boundaries when continuous variables were categorized<br>Not applicable.
		(*c*) If relevant, consider translating estimates of relative risk into absolute risk for a meaningful time period<br>Not applicable.

Other analyses	17	Report other analyses done—eg analyses of subgroups and interactions, and sensitivity analyses<br>Not applicable.
**Discussion**		
Key results	18	Summarise key results with reference to study objectives<br>Included in the Discussion on page 17.
Limitations	19	Discuss limitations of the study, taking into account sources of potential bias or imprecision. Discuss both direction and magnitude of any potential bias<br>Included in the Discussion on pages 19 and 20.
Interpretation	20	Give a cautious overall interpretation of results considering objectives, limitations, multiplicity of analyses, results from similar studies, and other relevant evidence<br>Included in the Discussion on pages 16,17,18,19 and 20.
Generalisability	21	Discuss the generalisability (external validity) of the study results<br>Included in the Discussion on page 20.
**Other information**		
Funding	22	Give the source of funding and the role of the funders for the present study and, if applicable, for the original study on which the present article is based<br>Included in page 21.

*Give information separately for exposed and unexposed groups.

Note: An Explanation and Elaboration article discusses each checklist item and gives methodological background and published examples of transparent reporting. The STROBE checklist is best used in conjunction with this article (freely available on the Web sites of PLoS Medicine at http://www.plosmedicine.org/, Annals of Internal Medicine at http://www.annals.org/, and Epidemiology at http://www.epidem.com/). Information on the STROBE Initiative is available at http://www.strobe-statement.org.

Table S2. Variables description.

<table>
<tr><th colspan="2">Variables</th><th>Type</th><th>Source</th><th>Link</th></tr>
<tr><td colspan="5">Demographic information</td></tr>
<tr><td colspan="2">Small Area Socioeconomic Index (IST)</td><td>Quantitative</td><td>Statistics Institute of Catalonia</td><td>https://www.idescat.cat/pub/?id=ist&lang=en</td></tr>
<tr><td colspan="5">Pre-stroke clinical</td></tr>
<tr><td colspan="2">Cardiovascular risk factors</td><td rowspan="3">Quantitative</td><td rowspan="3">Minimum Basic DataSet Primary Care</td><td rowspan="3">https://scientiasalut.gencat.cat/handle/11351/1121</td></tr>
<tr><td colspan="2">Funtional status</td></tr>
<tr><td colspan="2">Secondary cardiovascular prevention</td></tr>
<tr><td colspan="5">Stroke episode</td></tr>
<tr><td colspan="2">Transportation</td><td rowspan="3">Quantitative</td><td rowspan="3">Minimum Basic DataSet Emergencies</td><td rowspan="3">https://scientiasalut.gencat.cat/handle/11351/1120.3</td></tr>
<tr><td colspan="2">Stroke emergency visits</td></tr>
<tr><td colspan="2">Stroke type</td></tr>
<tr><td colspan="2">Stroke code Activation</td><td rowspan="3">Quantitative</td><td rowspan="3">Acute Stroke Online Information System (CICat)</td><td rowspan="3">https://aquas.gencat.cat/ca/fem/intelligencia-analitica/registre-cicat/resultats/</td></tr>
<tr><td colspan="2">Neuroimaging (CT or NMR)</td></tr>
<tr><td colspan="2">Reperfusion treatment</td></tr>
<tr><td colspan="5">Post-Stroke</td></tr>
<tr><td colspan="2">Home discharge</td><td>Quantitative</td><td>Minimum Basic DataSet Acute Care</td><td>https://scientiasalut.gencat.cat/handle/11351/1170</td></tr>
<tr><td colspan="2">Length of stay</td><td>Quantitative</td><td>Minimum Basic DataSet Intermediate Care</td><td>https://scientiasalut.gencat.cat/handle/11351/1126.4</td></tr>
<tr><td rowspan="2">Rehabilitation</td><td>Home</td><td rowspan="2">Quantitative</td><td>Minimum Basic DataSet Acute Care</td><td>https://scientiasalut.gencat.cat/handle/11351/1170</td></tr>
<tr><td>Hospital</td><td>Minimum Basic DataSet Intermediate Care</td><td>https://scientiasalut.gencat.cat/handle/11351/1126.4</td></tr>
<tr><td colspan="5">Dependece post-stroke</td></tr>
<tr><td colspan="2">Recognized Grades of Dependence</td><td>Qualitative</td><td rowspan="3">Department of Social Rights</td><td rowspan="3">https://dretssocials.gencat.cat/web/.content/03ambits_tematics/15serveissocials/estadistiques/mapa_serveis_socials/2022/arxius/mapa-serveis-socials-2022-prestacions-economiques-serveis-persones-dependencia.pdf</td></tr>
<tr><td colspan="2">Family caregiver</td><td>Quantitative</td></tr>
<tr><td colspan="2">External caregiver</td><td>Quantitative</td></tr>
</table>

Follow-up			
Follow-up visits	Quantitative	Project "Planifi.cat" for treating cronicity in Primary Care	https://si9sapics.wordpress.com/tag/planificat/
In-Hospital Death	Quantitative	Minimum Basic DataSet Acute Care	https://scientiasalut.gencat.cat/handle/11351/1170
